# Sleep Extension Improves Neurocognitive Functions in Chronically Sleep-Deprived Obese Individuals

**DOI:** 10.1371/journal.pone.0084832

**Published:** 2014-01-15

**Authors:** Eliane A. Lucassen, Paolo Piaggi, John Dsurney, Lilian de Jonge, Xiong-ce Zhao, Megan S. Mattingly, Angela Ramer, Janet Gershengorn, Gyorgy Csako, Giovanni Cizza, for the Sleep Extension Study Group

**Affiliations:** 1 Clinical Center, NIH, Bethesda, Maryland, United States of America; 2 Laboratory for Neurophysiology, Department of Molecular Cell Biology, Leiden University Medical Center, Leiden, The Netherlands; 3 Center for Neuroscience and Regenerative Medicine, Bethesda, Maryland, United States of America; 4 Intramural Research Program, NIDDK, NIH, Bethesda, Maryland, United States of America; 5 Section on Neuroendocrinology of Obesity, NIDDK, NIH, Bethesda, Maryland, United States of America; 6 Obesity Research Center, Endocrinology Unit, University Hospital of Pisa, Pisa, Italy; 7 Department of Laboratory Medicine, Clinical Center, NIH, Bethesda, Maryland, United States of America; Oregon Health & Science University, United States of America

## Abstract

**Background:**

Sleep deprivation and obesity, are associated with neurocognitive impairments. Effects of sleep deprivation and obesity on cognition are unknown, and the cognitive long-term effects of improvement of sleep have not been prospectively assessed in short sleeping, obese individuals.

**Objective:**

To characterize neurocognitive functions and assess its reversibility.

**Design:**

Prospective cohort study.

**Setting:**

Tertiary Referral Research Clinical Center.

**Patients:**

A cohort of 121 short-sleeping (<6.5 h/night) obese (BMI 30–55 kg/m^2^) men and pre-menopausal women.

**Intervention:**

Sleep extension (468±88 days) with life-style modifications.

**Measurements:**

Neurocognitive functions, sleep quality and sleep duration.

**Results:**

At baseline, 44% of the individuals had an impaired global deficit score (*t*-score 0–39). Impaired global deficit score was associated with worse subjective sleep quality (p = 0.02), and lower urinary dopamine levels (p = 0.001). Memory was impaired in 33%; attention in 35%; motor skills in 42%; and executive function in 51% of individuals. At the final evaluation (N = 74), subjective sleep quality improved by 24% (p<0.001), self-reported sleep duration increased by 11% by questionnaires (p<0.001) and by 4% by diaries (p = 0.04), and daytime sleepiness tended to improve (p = 0.10). Global cognitive function and attention improved by 7% and 10%, respectively (both p = 0.001), and memory and executive functions tended to improve (p = 0.07 and p = 0.06). Serum cortisol increased by 17% (p = 0.02). In a multivariate mixed model, subjective sleep quality and sleep efficiency, urinary free cortisol and dopamine and plasma total ghrelin accounted for 1/5 of the variability in global cognitive function.

**Limitations:**

Drop-out rate.

**Conclusions:**

Chronically sleep-deprived obese individuals exhibit substantial neurocognitive deficits that are partially reversible upon improvement of sleep in a non-pharmacological way. These findings have clinical implications for large segments of the US population.

**Trail registration:**

www.ClinicalTrials.gov NCT00261898. NIDDK protocol 06-DK-0036

## Introduction

According to the latest US surveys, 36% of the adult population is obese [1] and one third of adults report sleeping less than 6 h per night, substantially less than the recommended 7–9 h sleep per night [2]. Chronic sleep deprivation and obesity may be related in a bidirectional fashion, and they have similar consequences, including hypertension, diabetes, and cardiovascular disease [3], [4]. In addition, obesity and sleep deprivation have been linked to cognitive deficits.

Obese individuals exhibit deficits in executive functions, including mental flexibility, planning, problem solving, and display impulsivity [5]–[9], and tend to favor immediate reward *vs.* long-term gain [10]. Possibly, the “higher cortical functions” that control learning and executive function are no longer appropriately inhibiting feeding behavior in obese individuals. In turn, altered eating behavior in obesity may *cause* neurocognitive dysfunction; for example a high-fat or high-carbohydrate meal can disrupt hippocampal function [11].

Acute sleep deprivation predominantly interferes with attention and memory, while performance on more complex tasks is relatively intact [12]. One night of total sleep deprivation impaired attention in lean male volunteers [13], and habitual short sleep decreased memory function in adolescents [14]. A meta-analysis reported that sleeping less than 30 hours per week caused severe impairment in the clinical performance of medical care providers [15]. In addition, chronic sleep deprivation exacerbates symptoms of Attention Deficit Hyperactivity Disorder (ADHD) [16]. Furthermore, sleep extension can improve declarative memory in adolescents [17]. Interestingly, six days of partial sleep restriction increased the activation of food reward pathways –a putative pathway leading to weight gain in chronically sleep-deprived individuals [18].

Cognitive performance has never been evaluated in a sample of individuals who are both obese and chronically sleep deprived. Our goal was to characterize neurocognitive functions in this population and to prospectively assess reversibility of possible deficits with sleep extension achieved in a non-pharmacological way under real-life conditions.

## Methods

### Ethics Statement

The NIDDK Institutional Review Board approved the protocol and each individual gave written informed consent. This research was conducted according to the principles expressed in the Declaration of Helsinki.

### Study Design and Individuals

The *Sleep Extension Study* was a randomized, controlled, prospective study of sleep extension in chronically sleep-deprived obese individuals. Details have been provided elsewhere [19]. In brief, individuals were recruited by advertising for men and premenopausal women aged 18 to 50 years with a body mass index (BMI) between 30 and 55 kg/m^2^ who reported sleeping less than 6.5 hrs per night. Individuals self-identified their ethnicity as “black”, “white”, or “other”.

Out of 121 individuals with baseline neuropsychological evaluation, 72 were randomized to the Intervention Group and 49 to the Comparison Group. Individuals in the Intervention Group were coached to increase sleep duration up to 7.5 h per night, following a personalized sleep plan. Strategies included implementation of consistent bedtime routine, avoiding caffeine, alcohol, heavy meals and exercise prior to bedtime, creating an environment conducive to sleep, controlling bedroom light and temperature. The Comparison Group was asked to continue the existing short sleep habits. Sleeping habits were reviewed approximately every two months.

### Study Measurements

#### (A) Neurocognitive Battery

The battery of tests was administered in approximately 1.5 h between 2 pm and 6 pm in the following consistent order:

1) **Wechsler Abbreviated Scale of Intelligence (WASI)** (Psychological Corporation 1999, San Antonio, TX, USA): a brief test derived from the WAIS-III to estimate full scale IQ.

2) **Rey Complex Figure Test** [20]: assesses visuo-spatial construction, nonverbal memory, attention, and planning.

3) **California Verbal Learning Test (CVLT-II)** [21]: a list-learning task to evaluate verbal learning and memory.

4) **Grooved Peg Board Test (GPeg)** (Lafayette Instrument Company, Lafayette, IN, USA): a test of fine motor, manual dexterity.

5) **Wisconsin Card Sort Test (WCST)** [22]: a measure of executive function, emphasizing abstract reasoning, ability to use feedback and to shift cognitive sets.

6) **Trail Making Test (TMT) parts A and B** [23]: tests visual attention and task switching that requires visual scanning, sequencing, and visual motor speed.

7) **Verbal Fluency Test (FAS)** [24]: assesses phonemic fluency associated with executive functioning.

8) **Iowa Gambling Task (IGT)** [25]: a computer-based program developed to assess decision-making in a test that emulates gambling with varied cost versus payoff ratios.

These tests were grouped into four cognitive ability domains: a) Memory (Rey Delayed Recall, CVLT-II Short and Long Delay), b) Attention (Rey Immediate Recall, CVLT-II Sum, TMT-A), c) Motor skills (GPeg), and d) Executive function (TMT-B, WCST, FAS, IGT). The same battery of tests was administered at baseline and at the final follow up visit, except the WASI, which was only administered at baseline. The time interval between administrations of the tests would not be likely to produce practice effects [26].

#### (B) Anthropometrics and Body Composition

Height was measured using a wall mounted stadiometer (SECA 242, SECA North America East, Hanover, MD, USA) and weight was measured using a stand-on-scale in a hospital gown to the nearest 1/10th of a kg (SR555 SR Scales, SR Instruments, INC, Tonawanda, NY, USA). Circumference measurements were done using a non-stretch measuring tape in triplicate to the nearest mm. Waist circumference was measured at the uppermost lateral border of iliac crest at the end of a normal expiration. If this site could not be determined, the maximum circumference was measured at or near the level of the umbilicus. Neck circumference was measured at the minimal circumference with the head in the Frankfurt Horizontal Plane.

Abdominal visceral fat deposits were assessed by CT scans at the L2–L3 and L4–5 levels using a HiSpeed Advantage CT/I scanner (GE Medical Systems, Milwaukee, WI, USA) and analyzed on a SUN workstation using the MEDx image analysis software package (Sensor System, Sterling, VA, USA). Hypertension was defined as a blood pressure of ≥140/90 mmHg, and metabolic syndrome was determined by the NCEP ATP-III criteria [27].

#### (C) Subjective and Objective Sleep Measures

Sleep duration was assessed by two-week sleep diaries and by concomitant usage of wrist actigraphy (Actiwatch-64, Mini Mitter/Respironics/Philips, Bend, OR, USA) *via* recording gross locomotor activity in one-minute epochs. Sleep efficiency was the sleep time divided by the time spent in bed. The respiratory disturbance index (RDI; the number of (hypo)apneas per hour of sleep) was documented overnight by a portable validated screening device (Apnea Risk Evaluation System, Advanced Brain Monitoring Inc., Carlsbad, CA, USA) [28]. Daytime sleepiness was assessed by the Epworth Sleepiness Scale (ESS), a validated 8-item questionnaire with scores ranging from 0 to 24, with higher scores representing greater daytime sleepiness [29]. The Pittsburgh Sleep Quality Index (PSQI) is a validated 21-item questionnaire that quantifies subjective sleep quality [30]. PSQI scores range from 0 to 21 and higher scores indicate worse sleep.

#### (D) Clinical Laboratory Assessments

Plasma ACTH and serum cortisol were determined by immunochemiluminescence methods (Immulite 2000 and 2500, Siemens Health Diagnostics, Deerfield, IL, USA). Total plasma ghrelin was measured with a sandwich ELISA kit measuring both intact and des-octanoyl forms with the lowest level of detection at 50 pg/mL for total ghrelin (Millipore, Billerica, MA, USA). The intra- and inter-assay CV's were 1.96% and 7.8%, respectively. Urine catecholamines and urinary free cortisol (UFC) were measured in 24 h-collections with high-performance-liquid chromatography, and liquid chromatography-tandem mass spectrometry, respectively.

### Statistical Analysis

Normality was determined by Q-Q plots. For normally distributed variables and for skewed variables, mean and standard deviation (SD) and median and interquartile range (IQR; 25^th^ and 75^th^ percentile) were computed, respectively.

All raw tests scores (TMT, GPeg, FAS) were converted to demographically adjusted *t*-scores, using Heaton norms [31] or test-specific norms, ranging from 0 to 100 (mean (SD): 50 (10)). A *t*-score below 40 was considered impaired; 40–44 was below average; 45–54 was average; and ≥55 was above average. Individual Rey scores were used only if the figure was copied well (Rey Copy *t*-score >39).

Raw Global Deficit Scores (GDS) were calculated by adding penalty points for the impairment category per separate test (*t*-score <20 = 5 points; *t*-score 20-24 = 4 points; t-score 25-29 = 3 points; *t*-score 30-34 = 2 points; *t*-score 35-39 = 1 point; and *t*-score >39 = 0 points), and dividing this number by the number of tests analyzed per individual. Domain Deficit Scores (DDS) were similarly computed for the tests of the specific neurocognitive domain. Raw GDS/DDS were then transformed into *t*-scores [31]. The GDS/DDS were considered “impaired” if the *t*-score was <40. This cut-off value has 69% sensitivity, and 91% specificity for diagnosing impairment [32].

Independent *t*-tests and Mann-Whitney U tests were used to compare characteristics between individuals with a GDS/DDS *t*-score <40 *vs.* a *t*-score of ≥40. Statistical tests used to compare changes between baseline and follow-up included paired Student *t*-tests and Wilcoxon tests for skewed variables.

A multivariate mixed model using both baseline and follow-up data determined which variables were associated with GDS, after accounting for repeated measurements. Statistical significance was defined as p<0.05.

## Results

### Baseline Neuropsychological, Demographic, Anthropometric, Sleep, and Hormonal Characteristics According to Global Deficit Score and Domain Deficit Score

The mean age of the 121 individuals with neuropsychological testing at baseline was 41.1±7.0 years, most were women (76%) and black (60%). They had 15.8±2.5 years of education, an IQ of 112.8±12.0, and a BMI of 38.6±6.4 kg/m^2^. Sleep duration by diaries averaged 6 h and 25 min (385±48 min/night), whereas sleep duration by actigraphy monitors was approximately 30 min shorter (356±49 min/night). Seven percent of the individuals smoked, 13% had hypertension, and 26% had metabolic syndrome.

**Figure 1:**
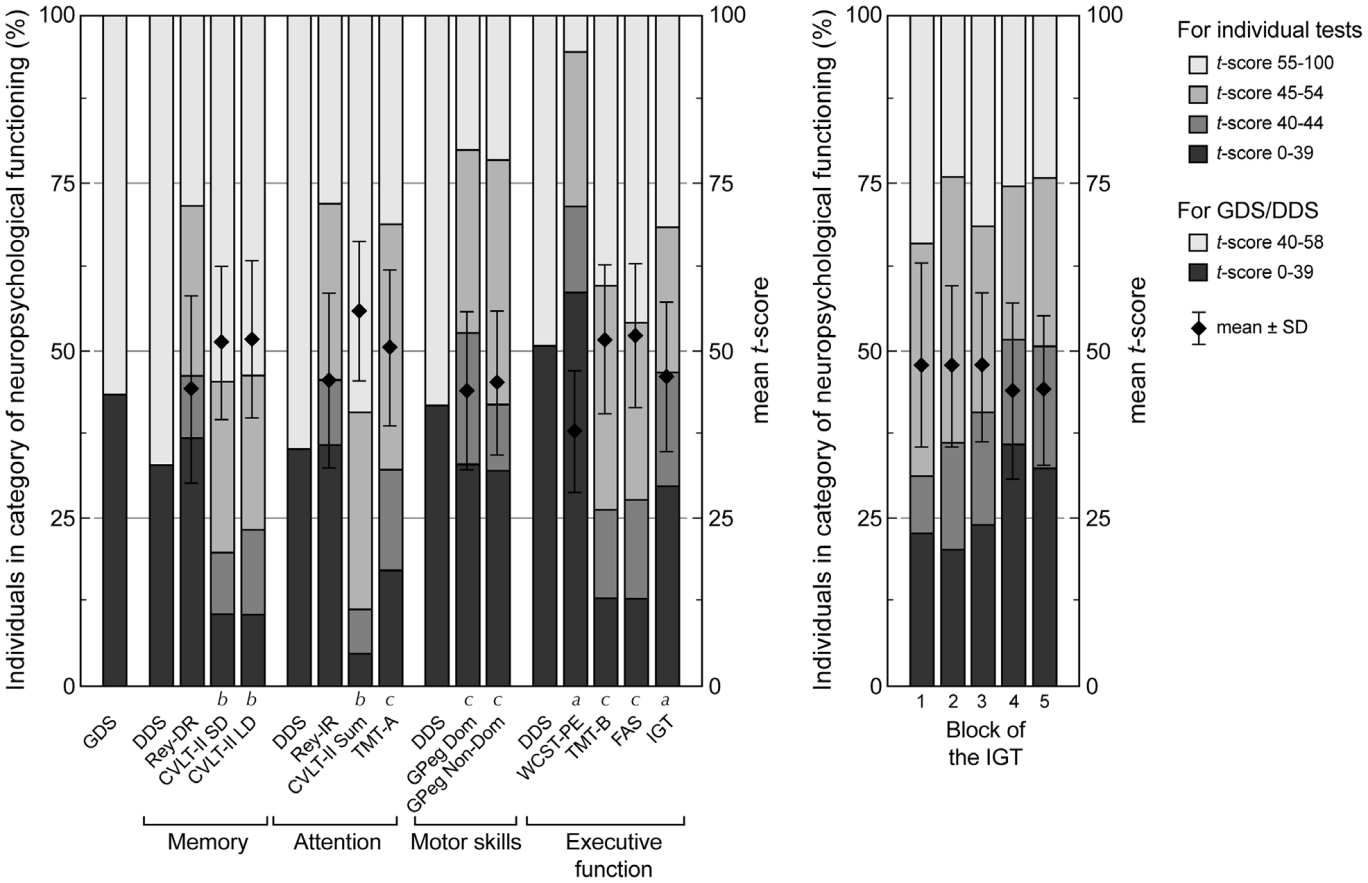
Neurocognitive function at baseline. *Left Figure*: Results of deficit scores and individual neurocognitive tests. *Right Figure*: Results of the IGT per subsequent block of the test. The left vertical axis depicts the percentage of individuals in the following categories of neuropsychological function levels: impaired (*t*-score 0–39), below average (*t*-score 40–44), average (*t*-score 45–54), and above average (*t*-score 55–100). The right y-axis shows the mean±SD of the *t*-scores for each separate test. Mean and SD for deficit scores are not shown, because these variables exhibited a skewed distribution. Memory was tested by Rey-DR, the CVLT-II SD and the CVLT-II LD. Attention was tested by the CVLT-II Sum and by the TMT-A. Motor skills were assessed by the GPeg Dom and the GPeg Non-Dom. Executive functions were tested by the WCST, the TMT-B and the IGT. For all *t*-score computations, age was taken into account. In addition, ^a^years of education were taken into consideration for the *t*-scores for the WCST and IGT, and ^b^gender was taken into account for the CVLT scores. ^c^Education, race, and gender were all taken into account for *t*-scores of the TMT, GPeg, and FAS. *Abbreviations*: GDS  =  Global Deficit Score, DDS  =  Domain Deficit Score, Rey-DR  =  Rey Complex Figure Test Delayed Recall, CVLT-II SD  =  California Verbal Learning Test II Short Delay, CVLT-II LD  =  California Verbal Learning Test II Long Delay, CVLT-II Sum  =  California Verbal Learning Test II Sum Score, TMT-A =  Trail Making Test Part A, TMT-B =  Trail Making Test Part B, GPeg Dom  =  Grooved Peg Board Test Dominant Hand, GPeg Non-Dom  =  Grooved Peg Board Test Non-Dominant Hand, WCST  =  Wisconsin Card Sort Test, IGT  =  Iowa Gambling Task.

Fig 1 depicts the baseline *t*-score for the global neuropsychological function, as well as sub-categorized in the memory, attention, motor skills, and executive functions domains. The mean GDS was 41.0±7.0, interpreted as below average. Memory was impaired in 33%, attention in 36%, motor skills in 42%, and executive function in 51% of the individuals.

Table 1 reports demographic, anthropometric, sleep, and hormonal characteristics divided in two groups according to their GDS score. Approximately 44% of individuals had an impaired (0–39) *t*-score. These individuals had worse sleep quality by PSQI, lower urinary dopamine, and tended to have lower urinary norepinephrine and lower UFC. Demographic and anthropometric characteristics of the two groups were similar. Specific features that differed at a p level of ≤0.1 for each of the cognitive domains examined are also shown.

**Table 1:** Baseline characteristics of individuals according to Global Deficit Score and Domain Deficit Score categories.

Global Deficit Score			
	*t*-score 0–39 (N = 53)	*t*-score 40–58 (N = 68)	*P* value
*Demographic*			
Age (yrs)	41.8 (6.3)	40.4 (7.5)	0.27
Sex (% female)	83	71	0.11
Race (White/Black/Other in %)	62/36/2	57/35/7	0.38
Education (yrs)	15.7 (2.3)	15.8 (2.7)	0.73
Smokers (%)	8	6	0.75
Hypertension (≥140/90 mmHg) (%)	16	13	0.62
Metabolic syndrome (%)	26	30	0.63
*Anthropometric*			
BMI (kg/m^2^)	37.8 (6.0)	38.9 (6.6)	0.34
Waist circumference (cm)	113.2 (12.4)	115.6 (13.7)	0.88
Neck circumference (cm)	38.6 (4.0)	39.9 (4.3)	0.71
Abdominal visceral fat (cm^3^)	319.0 (152.4)	356.7 (171.7)	0.98
*Sleep*			
Sleep duration by PSQI (min)	334 (62)	338 (45)	0.73
Sleep duration by diary (min)	383 (43)	387 (52)	0.70
Sleep duration by actigraphy (min)	351 (47)	345 (50)	0.48
Percent sleep efficiency	79.0 (7.3)	80.4 (5.7)	0.27
RDI (events/h)	7 (2–18)	8 (3–13)	0.65
Daytime sleepiness (ESS score)	8.1 (4.8)	8.4 (4.4)	0.71
Subjective sleep quality (PSQI score)	8.9 (2.9)	7.7 (2.7)	**0.04**
Abnormal subjective sleep quality (PSQI score >4) (%)	93	76	**0.02**
*Hormonal*			
Plasma ACTH (pg/mL)	18.2 (13.0–26.1)	18.9 (14.3–27.4)	0.48
Serum cortisol (µg/dL)	9.5 (3.8)	9.5 (4.3)	0.99
Urinary dopamine (µg/24 h urine)	239 (169–292)	276 (230–341)	**0.001**
Urinary epinephrine (µg/24 h urine)	3.4 (2.1–4.9)	4.5 (2.3–6.6)	0.24
Urinary norepinephrine (µg/24 h urine)	38.0 (28.0–53.0)	44.5 (35.0–59.0)	0.07
Urinary free cortisol (µg/24 h urine)	17.3 (12.0–23.3)	19.5 (14.5–28.3)	0.07
Plasma total ghrelin (pg/mL)	302.6 (199.3)	254.7 (124.6)	0.12

Domain Deficit Score			
	*Memory t*-score 0-39 (N = 40)	*Memory t*-score 40-58 (N = 81)	*P* value
Sleep efficiency (%)	78.1 (6.7)	80.6 (6.2)	0.06
Urinary dopamine (µg/24 h urine)	228 (146–310)	269 (228–325)	**0.006**
Urinary norepinephrine (µg/24 h urine)	38.0 (26.0–52.0)	44.0 (35.0–35.0)	**0.04**
Urinary free cortisol (µg/24 h urine)	14.5 (10.3–20.0)	20.8 (15.0–28.5)	**0.01**
	*Attention t*-score 0-39 (N = 43)	*Attention t*-score 40-58 (N = 78)	*P* value
Sleep efficiency (%)	78.0 (6.9)	80.8 (6.0)	**0.02**
Urinary dopamine (µg/24 h urine)	241 (183–294)	276 (223–338)	**0.01**
Urinary norepinephrine (µg/24 h urine)	37.5 (23.8–53.0)	45.0 (36.0–59.0)	**0.02**
Urinary free cortisol (µg/24 h urine)	16.0 (10.0–24.0)	19.0 (15.0–28.0)	**0.02**
	*Motor Skills t*-score 0–39 (N = 51)	*Motor Skills t*-score 40–58 (N = 70)	*P* value
Sleep efficiency (%)	78.1 (7.4)	81.1 (5.4)	**0.02**

Values are means (SD) for normally distributed variables, percent, or median (interquartile range, IQR) for skewed variables. *t*-tests were performed for continuous variables (skewed variables were log transformed before computations) and Chi-squared tests were used for categorical variables. *Abbreviations*: BMI  =  Body Mass Index, PSQI  =  Pittsburgh Sleep Quality Index, RDI  =  Respiratory Disturbance Index, ESS  =  Epworth Sleepiness Scale, ACTH  =  Adrenocorticotropic Hormone. *SI conversion factors*: To convert ACTH to pmol/L, multiply values by 0.22; to convert cortisol to nmol/L, multiply values by 27.588; to convert dopamine to nmol/d, multiply values by 6.528; to convert epinephrine to nmol/d, multiply values by 5.459; to convert norepinephrine to nmol/d, multiply values by 5.911; to convert free cortisol to nmol/d, multiply values by 2.759; to convert total ghrelin to pmol/L, multiply values by 0.296.

Individuals with an impaired memory had significantly lower urinary dopamine, norepinephrine, and UFC levels and a tendency for lower sleep efficiency. Individuals with impaired attention had lower sleep efficiency, and lower urinary dopamine, norepinephrine, and UFC levels. Participants with impaired motor skills had lower sleep efficiency. There were no differences at the p ≤0.1 level for participants with impaired *vs.* unimpaired executive functions.

### Changes between the Two Neuropsychological Evaluations

Of the 121 original individuals with baseline neuropsychological evaluation, 74 individuals (34 from the Comparison Group and 40 from the Intervention Group; 51F/23M) had another neuropsychological evaluation 468±88 days later (median 436, IQR 412–496 days) (Table 2). Group allocation and GDS category were similar between the 74 individuals who completed the study and the 47 individuals who did not (p = 0.836 and p = 0.240, respectively). Because there were no significant differences in neuropsychological functions between the Comparison and the Intervention Groups either at the initial or at the final evaluation, results from the two groups were combined together.

**Table 2:** Changes in neurocognitive functions between the first and the final follow-up evaluation.

	Baseline (N = 74)^a^	Final follow-up (N = 74)^a^	*P* value	
**Global Deficit Score and Domain Deficit Score**				
Global Deficit Score	43.5 (9.2)	46.6 (9.0)	**0.001**	
Memory Domain Deficit Score	47.7 (14.3)	50.9 (11.3)	0.07	
Attention Domain Deficit Score	47.6 (11.3)	52.5 (10.2)	**0.001**	
Motor Skills Domain Deficit Score	45.6 (14.6)	47.5 (14.7)	0.26	
Executive function Domain Deficit Score	42.8 (9.8)	45.1 (11.0)	0.06	
**Specific Neurocognitive Test ** ***t*** **-Scores**				
*Rey Complex Figure Test*				
Copy (N = 73)	44.4 (9.0)	43.3 (8.8)		0.26
Immediate Recall^b^ (N = 49)	47.9 (12.7)	52.3 (12.4)		**0.002**
Delayed Recall^b^ (N = 49)	45.6 (14.1)	51.1 (12.2)		**<0.001**
*California Verbal Leaning Test*				
Long Delay (N = 72)	52.1 (11.9)	53.5 (11.4)		0.12
Short Delay (N = 71)	51.5 (12.1)	53.1 (11.4)		0.08
Sum (N = 72)	55.4 (11.3)	57.9 (12.6)		**0.01**
*Grooved Peg Board Test*				
Dominant Hand (N = 69)	44.3 (11.8)	45.0 (11.9)		0.68
Non-Dominant Hand (N = 72)	44.8 (10.0)	46.1 (10.6)		0.17
Wisconsin Card Sort Test (Perseverative Errors; N = 72)	37.6 (9.1)	38.4 (7.7)		0.38
Trail Making Test part A (N = 73)	50.1 (12.0)	52.5 (12.7)		0.15
Trail Making Test part B (N = 72)	52.2 (11.6)	53.9 (11.8)		0.30
Verbal Fluency Test (N = 73)	52.4 (10.8)	52.9 (10.7)		0.60
Iowa Gambling Task (N = 48)	47.0 (11.2)	50.8 (11.5)		**0.04**

Values are means (SD).

^a^ Number of individuals per time point unless otherwise.

^b^ Scores for Immediate and Delayed Recall were only included if the Copy Score was at least 40, so that memory function was assessed in a more reliable manner. *Abbreviations*: GDS  =  Global Deficit Score; DDS  =  Domain Deficit Score.

The following changes were observed: the GDS improved by approximately 7%; attention improved by 10%; memory and executive functions tended to improve by 7% and 5%, respectively, whereas motor skills did not change. Individual tests that improved over time included the Rey Delayed Recall (memory), Rey Immediate Recall and CVLT-Sum (attention), and the IGT (executive functions) (Table 2).

Subjective sleep quality improved by 24%, self-reported sleep duration increased by 11% by PSQI and by 4% by diaries, respectively, and sleepiness tended to improve. Serum cortisol increased by approximately 17% (Table 3). There were no significant changes in urinary dopamine, epinephrine and norepinephrine, UFC, and plasma total ghrelin. Of note, each of the characteristics shown in Table 2 and Table 3 were similar between “completers” and “non-completers” (data not shown) with the exception of UFC that was significantly lower in completers *vs.* non-completers (19.3±12.6 *vs.* 24.8±15.9 µg/24 h urine, p = 0.038).

**Table 3:** Changes in sleep and hormonal parameters between the baseline and final follow up evaluation.

Parameters	Baseline	Final follow-up	*P* value	
*Sleep parameters*				
Subjective sleep quality (PSQI score; N = 64)	8.0 (2.8)	6.1 (2.4)	**<0.001**	
Sleep duration by PSQI (min; N = 64)	336 (60)	372 (72)	**<0.001**	
Sleep duration by diary (min; N = 40)	388.3 (53.1)	404.6 (48.5)	**0.04**	
Daytime sleepiness (ESS score; N = 63)	8.8 (4.8)	8.0 (4.5)	0.10	
*Hormonal parameters*				
Serum cortisol (µg/dL; N = 67)	8.7 (3.8)	10.2 (4.8)	**0.02**	
Urinary dopamine (µg/24 h urine; N = 69)	256 (199–317)	241 (188–331)	0.53	
Urinary epinephrine (µg/24 h urine; N = 69)	3.3 (2.0–4.8)	3.8 (2.3–5.5)	0.11	
Urinary norepinephrine (µg/24 h urine; N = 69)	38.5 (28.5–56.5)	40.0 (33.0–55.5)	0.35	
UFC (µg/24 h urine; N = 69)	15.0 (10.0–21.0)	16.0 (10.0–26.0)	0.76	
Plasma total ghrelin (pg/mL; N = 67)	268.8 (148.8)	292.1 (204.6)	0.32	

Values are means (SD) for normally distributed variables and median (interquartile range, IQR) for skewed variables. *t*-tests were performed (skewed variables were log transformed before computations). *Abbreviations*: PSQI  =  Pittsburgh Sleep Quality Index, ESS  =  Epworth Sleepiness Scale, UFC  =  Urinary Free Cortisol. *SI conversion factors*: To convert cortisol to nmol/L, multiply values by 27.588; to convert dopamine to nmol/d, multiply values by 6.528; to convert epinephrine to nmol/d, multiply values by 5.459; to convert norepinephrine to nmol/d, multiply values by 5.911; to convert free cortisol to nmol/d, multiply values by 2.759; to convert total ghrelin to pmol/L, multiply values by 0.296.

### Relationships between Neurocognitive Function by Domain Deficit Score, Obesity, Sleep and Urinary Stress Hormones

Five variables were included in the model, sleep quality by PSQI and sleep efficiency, and UFC, urinary dopamine and plasma total ghrelin, together accounting for 1/5 of the variability in global cognitive functions (Table 4; Model A). The effect size was of clinical significance; as an example, an improvement in sleep quality of 1 unit would improve the GDS by 0.64 units, whereas an improvement in sleep efficiency of 1 unit would improve the GDS by 0.25 units. Adjustment by age increased the accounted variability from 22% to 28% (Model B), whereas adjustments by gender, or ethnicity did not substantially change the explained variability (data not shown). Each model included the group allocation and follow-up time as covariates.

**Table 4:** Mixed models for Global Deficit Score.

*Dependent Variable*: Global Deficit Score	*Model A:unadjusted*	*Model B:adjusted by age*
*Intercept*	22.5 (8.3) **(P = 0.008)**	12.4 (9.2) (P = 0.18)
*Goodness of fit*	R^2^ = 0.224 **(P<0.001)**	R^2^ = 0.280 **(P<0.001)**
PSQI score	−0.64 (0.24) **(P = 0.01)**	−0.70 (0.24) **(P = 0.005)**
Sleep efficiency (%)	0.25 (0.10) **(P = 0.01)**	0.23±0.10 **(P = 0.02)**
UFC (µg/24 h urine)	0.09 (0.05) (P = 0.09)	0.10 (0.05) (P = 0.06)
Urinary dopamine (µg/24 h urine)	0.02 (0.01) **(P = 0.001)**	0.03 (0.01) **(P<0.001)**
Plasma total ghrelin (pg/mL)	−0.01 (0.01) (P = 0.07)	−0.01 (0.04) **(P = 0.02)**
Group (0 = Comparison, 1 = Intervention)	−0.41 (1.56) (P = 0.79)	−0.66 (1.51) (P = 0.66)
Follow-up time (days)	0.0020 (0.0024) (P = 0.41)	0.0020 (0.0025) (P = 0.42)
Age (yrs)		0.27 (0.11) **(P = 0.02)**

Mean (SD) of the regression coefficient is depicted. The statistical model was fit as follows: at first, five candidate variables were preselected based on physiology. These included hormones, anthropometric and body composition features, glucose metabolism and sleep features. These variables were screened in bivariate analyses with the GDS score as dependent variable, using a *P* value less than 0.2, in order to avoid rejecting potentially important variables. Of note, the variables included in the model were also checked for multicolinearity by using the Variance Inflation Factor (VIF) index and found to be unrelated to each other (highest VIF = 1.6 where a value of 5 or higher is the conventional threshold used for multicolinearity). *Abbreviations*: PSQI  =  Pittsburgh Sleep Questionnaire Index, UFC  =  Urinary Free Cortisol. *SI conversion factors*: To convert cortisol to nmol/L, multiply values by 27.588; to convert dopamine to nmol/d, multiply values by 6.528; to convert total ghrelin to pmol/L, multiply values by 0.296.

## Discussion

To our knowledge, this is the first prospective report of neurocognitive functions in chronically sleep-deprived obese men and women. A large percentage of individuals displayed neurocognitive deficits of clinical relevance, even though this sample was otherwise relatively healthy, with few smokers and few individuals with hypertension. Furthermore, individuals with more accentuated deficits had worse sleep quality and sleep efficiency, and a distinct hormonal profile characterized by lower urinary catecholamines and lower UFC. In a multivariate model, approximately 1/5 of the variability in the GDS, a parameter known to be influenced by many different factors, was accounted for by sleep quality and efficiency, as well as UFC, urinary dopamine and plasma total ghrelin. When testing was repeated 14 to 15 months later, improvements in sleep achieved in a non-pharmacological fashion were accompanied by ameliorations in neurocognitive scores. These findings are strongly suggestive of a contributory role of sleep deprivation for the cognitive deficits observed. More importantly, they indicate that some of these deficits may be ameliorated by sleep.

Sleep duration as assessed by actigraphy monitors and sleep duration by diaries were different in our study. At baseline, sleep duration by actigraphy was approximately 30 min shorter than sleep duration by diaries. Sleep duration usually is estimated by self-reported sleep in epidemiological studies, by actigraphy in smaller studies, and by polysomnography (PSG) in clinical studies. No method is without limitations in determining sleep duration; even PSG, which is considered the “gold standard”, may interfere with sleep duration the night of the testing because of its “invasiveness”. The degree of difference and its direction, with longer sleep duration with diaries, was similar to what previously reported [33]. Of note, the high correlation (0.90) between actigraphy and PSG observed in lean healthy subjects may worsen in subjects with sleep apnea because of limb movements during hypocapnia [34]. Therefore, in a sample of obese subjects, many with sleep apnea, actigraphy may be less reliable than in lean subjects.

Obese subjects often have impairments in the executive domain [5], [8], [10], [11], while sleep deprivation decreases attention and impairs processing speed [12], [14], [16], [35]. Compared to other studies of obese adults, our participants had worse scores on the TMT-B (all tests of the executive domain) [35], but similar scores at the IGT or the WCST [8], [10], [36], [37]. Medical residents that habitually sleep less than six hours were quicker on the TMT-A and TMT-B (attention and executive function, respectively), and scored better on the CVLT-LD (memory) than our participants [37]. As in these studies scores were not corrected for demographic characteristics, differences with our findings should be interpreted with caution.

Sleep quality was worst in participants with memory, attention and motor domains impairments. This is likely independent of sleep apnea, since the RDI was similar between participants with impaired *vs.* normal cognitive function. Deficits in memory and executive functions have been reported in men with sleep apnea [38]. Another comparison to our participants is patients with chronic non-restorative sleep, in which memory function on the Rey test was correlated with sleep efficiency [39]. Patients with restless leg syndrome who experience decreased sleep efficiency also have impairments in executive functions [40].

Sleep loss affects attention span. Because executive functions are impaired in obese individuals, we hypothesize that the compensatory ability of brain areas devoted to attention may be limited in obese, sleep-deprived individuals, resulting in a larger impairment compared to non-obese, sleep-deprived individuals. In our cohort, we found no relationship between adiposity and neurocognitive functions. This lack of association may be due to a “floor effect” –after a certain degree of obesity, cognitive functions may no longer be affected. Obese individuals with a mean BMI of 51 kg/m^2^ scored worse than our individuals on the Rey-test and on the WCST, indicating that further cognitive impairment may occur when obesity is extreme [5]. The observed decrease in attention and decision-making ability in obese, sleep-deprived individuals may increase their risk of accidents. Medical residents, shift workers, and truck drivers are at higher risk for errors because of sleep deprivation. Thus, obese, sleep-deprived individuals may be similarly prone to accidents.

Impairments in memory and attention were associated with lower UFC, and lower 24 h urinary dopamine and norepinephrine. Brain dopaminergic and noradrenergic systems modulate cognition [41], [42]. Since the stress response has a U-shaped curve, suboptimal levels of stress hormones may impair cognitive functions.

Plasma levels of the appetite stimulant ghrelin were inversely related to the GDS in our study participants (Table 4). This effect would be however of a modest clinical size: a 50-point increase in ghrelin would translate to only a decrease of 0.5 point in GDS. This finding may be of mechanistic interest, as the ghrelin system, in addition to its known role on appetite control, is involved in in neuroprotection, learning, and memory consolidation [43].

Study merits included the prospective, long-term characterization of a large cohort assembled *ad hoc*. Sleep duration was assessed with several complementary methods. However, the study design did not allow for discerning how much of the cognitive deficits could be attributed to chronic sleep deprivation *vs.* obesity. The presence of a group of lean, sleep-deprived individuals, and a group of obese, non-sleep deprived would have allowed determination of the effect of obesity *vs.* sleep deprivation on cognitive functions. Finally, loss to follow-up was approximately 40% in this challenging study, which is what is usually observed in prospective studies of obese subjects.

In summary, this is the first demonstration of sleep extension in a real life situation in chronically sleep-deprived obese individuals. This population exhibited neurocognitive deficits that were partially reversible; self-reported sleep quality, duration and sleepiness, all improved to a clinically meaningful extent as well. These improvements were achieved in a non-pharmacological way in a real life situation and were sustained over a long time. Approximately 86 million adult Americans are obese, 40 million US workers report an average sleep duration of <6 h according to the CDC. Our findings have clinical implications for vast segments of the US population with obesity, and sleep deprivation. Further research is needed to dissect the relative role of chronic sleep deprivation *vs.* obesity on cognitive functions. Prospective studies of obese individuals undergoing bariatric surgery may be warranted.
